# Effectiveness of a Physiotherapy Stress-Management Protocol on Cardiorespiratory, Metabolic and Psychological Indicators of Children and Adolescents with Morbid Obesity

**DOI:** 10.3390/children12081010

**Published:** 2025-07-31

**Authors:** Pelagia Tsakona, Alexandra Hristara-Papadopoulou, Thomas Apostolou, Ourania Papadopoulou, Ioannis Kitsatis, Eleni G. Paschalidou, Christos Tzimos, Maria G. Grammatikopoulou, Kyriaki Tsiroukidou

**Affiliations:** 1 Department of Physiotherapy, International Hellenic University, 57400 Thessaloniki, Greece; alekpap@phys.teithe.gr (A.H.-P.); apostolouthomas@ihu.gr (T.A.); raniapapadopoulouph2@gmail.com (O.P.); 2 Medical School, Aristotle University of Thessaloniki, University Campus, 54624 Thessaloniki, Greece; ikitsatis@auth.gr; 3 Endocrine Unit, 3rd Department of Pediatrics, Hippokration General Hospital of Thessaloniki, Aristotle University of Thessaloniki, 54124 Thessaloniki, Greece; elenipasxalidou99@gmail.com (E.G.P.); ktsiroukidou@gmail.com (K.T.); 4 Northern Greece Statistics Directorate, Hellenic Statistical Authority, 218 Delfon Str., 54646 Thessaloniki, Greece; ctzimos@gmail.com; 5 Immunonutrition Unit, Department of Rheumatology and Clinical Immunology, Faculty of Medicine, School of Health Sciences, University of Thessaly, Biopolis, 41110 Larissa, Greece; mgrammat@uth.gr

**Keywords:** weight loss, aerobic exercise, anxiety, cardiorespiratory fitness, insulin sensitivity, body fat, severe obesity, clinical trial

## Abstract

**Background:** Chronic stress in childhood and adolescence leads to excessive cortisol secretion, adipokines production and obesity with all the negative mental and physical effects on the health of individuals and adulthood. **Objectives:** The aim of the present non-randomized controlled trial was to investigate the effect of a stress management protocol with diaphragmatic breathing (DB) and physiotherapy exercise on stress, body composition, cardiorespiratory and metabolic markers of children and adolescents with morbid obesity. **Methods**: The study included 31 children and adolescents (5–18 years old) with morbid obesity (22 in the intervention arm and 9 controls). All participants completed anxiety questionnaires and a self-perception scale. Forced expiratory volume in the first second (FEV1), forced vital capacity (FVC), blood pressure (BP) and SpO_2_ were measured. Fasting glucose, uric acid, triglycerides, HbA1c, (AST/SGOT), (ALT/SGPT), HDL, LDL, insulin, ACTH, cortisol, HOMA-IR, 17-OH, S-DHEA, SHBG were assessed, and anthropometric measurements were also performed. **Results:** In the intervention group, 4 months after the treatment, an improvement was noted in the BMI, BMI z-score, waist-to-height ratio, FEV1, SpO_2_, pulse and systolic BP. HDL increased, ALT/SGPT and insulin resistance improved. Positive changes were observed in temporary and permanent stress and self-esteem of children in the intervention group, including anxiety, self-perception, physical appearance, etc. **Conclusions:** A combined exercise and DB protocol has a positive effect on stress, by improving body composition, reducing insulin resistance, and ameliorating physical and mental health and quality of life of pediatric patients with morbid obesity.

## 1. Introduction

The prevalence of childhood and adolescent obesity has increased significantly worldwide in less than a generation [1,2]. In childhood and adolescence, overweight and obesity lead to obesity in adulthood and expose children to a high risk of serious comorbidity, chronic diseases and mortality [3,4] thus justifying the need for early detection, orientation and treatment of the problem, especially in the ages where the problem is concentrated globally. This is supported by many studies showing an increase in the prevalence of childhood obesity worldwide and in Greece [5], also highlighting the importance of preventive interventions for obesity [6,7]. The treatment of children and adolescents with obesity is a difficult process, requiring many changes in the person’s life and psychological support [8,9]. Children with obesity have much higher rates of obstructive sleep apnea syndrome than children of normal weight, which leads to alveolar hypoventilation, decreased oxygen saturation, harmful effects on the central nervous and cardiovascular systems, metabolic syndrome, inability to concentrate in school, poor academic performance, behavioral problems and generally, poor development [10,11,12,13]. Additionally, 25% of children and adolescents with obesity suffer from hypertension [14]. The risk of elevated blood pressure (BP) persists in children with obesity as they progress into adulthood [15].

The stress system evolved to enable humans to escape from situations that threaten their survival, by activating various metabolic pathways. Multiple mechanisms exist, by which stress affects the development and maintenance of obesity [16]. Exposure to stress during childhood is associated with the development of obesity, although we do not yet fully know the exact metabolic mechanisms that cause it [17]. Stress in the first years of life (fetal, childhood, adolescence) is of particular importance. These specific periods are highly sensitive to stressful stimuli. The autonomic nervous system (ANS) plays a central role in the body’s response to stress. According to research, chronic stress in childhood is an important factor contributing to the development of obesity, as it dysregulates the endocrine system, the ANS and the immune system, leading to low-intensity inflammation [18]. Stress management could possibly aid weight loss, improve well-being and metabolic markers in children with obesity.

There is limited research on interventions carried out on children and adolescents with morbid obesity with the aim of improving their stress and quality of life (QoL). A randomized controlled trial involving a 6-month high-intensity aerobic exercise program and 2-year follow-up conducted in the Netherlands [19], revealed a significant improvement in the body mass index (BMI) z-score post-intervention; however, these favorable results were not maintained in the long term. In another trial conducted in adolescents with obstructive sleep apnea and severe obesity, lifestyle modification and long-term aerobic exercise (walking, swimming, cycling, climbing, and group games), resulted in body weight loss and improved cardiorespiratory fitness in all participants [20].

Two studies were conducted on Greek children with overweight and obesity involving 8-week stress management protocols, with a frequency of once per day, where diaphragmatic breathing (DB), muscle relaxation, guided imagery and cognitive restructuring, a diet program and physical activity were applied to the individuals in the intervention group of both studies. The results of the first study revealed an improvement in depression scales, anxiety, QoL and BMI of the children in the intervention arm [21], whereas the second study showed that stress management protocols can improve anthropometric indicators (waist-hip ratio), physical activity levels, school performance, as well as eating habits [22].

Children with obesity, and in particular those with morbid obesity, often encounter breathing problems due to increased body weight (e.g., reduced diaphragmatic function, dyspnea on exertion, sleep apnea). DB can improve lung function, reduce dyspnea and increase the capacity for physical activity. Obesity is also often associated with increased stress, anxiety and low self-esteem in children. DB is known to activate the parasympathetic nervous system, reducing stress and promoting relaxation [23,24]. Furthermore, difficulty in breathing limits children’s participation in physical activity, creating a vicious cycle. If DB improves endurance and respiratory performance, children may participate more actively in physical activities. Most interventions for stress management in children with obesity focus on diet and exercise. DB consists of an easy, inexpensive and applicable method that can be used in addition to other strategies. Although DB has been studied in adults and patients with respiratory problems [25], no studies have been conducted in children with morbid obesity and the effect of DB on stress management and QoL has never been assessed, indicating a knowledge gap.

The present non-randomized controlled clinical trial aimed to evaluate the effects of a DB and aerobic therapeutic exercise program on stress management and QoL in children with morbid obesity.

## 2. Materials and Methods

### 2.1. Participants Recruitment and Characteristics

The study population was recruited from the Pediatric and Adolescent Obesity Outpatient Clinic of the Pediatric Endocrinology Unit, situated at the 3rd University Pediatric Clinic of the Aristotle University of Thessaloniki, severely obese children and adolescents with a BMI > 120% of the 95th percentile.

**Figure 1:**
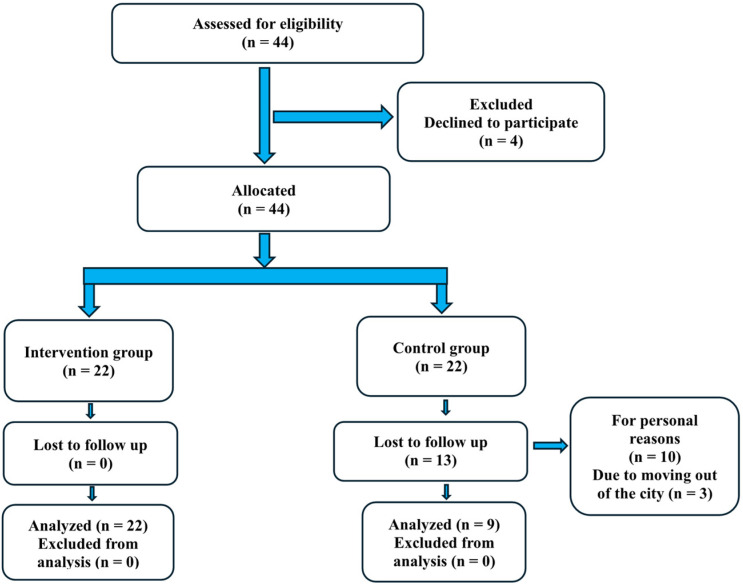
Diagram of the study’s participants selection process.

A total of 48 children and adolescents were initially selected from the hospital records. After communication and information regarding the purpose of the study, 44 individuals aged 5–18 years agreed to participate, who were divided into two equal groups: (a) intervention and (b) control arm (Figure 1).

A total of 13 children from the control group left the study before its completion, 10 for personal reasons and an additional 3 due to moving to other cities. Thus, a total of 31 children and adolescents with morbid obesity, aged 5–18 years participated in the study. The intervention group consisted of 22 children and the control group of 9 children. In terms of gender distribution, the intervention arm consisted of 12 boys and 10 girls (percentage 54.5% and 45.5%, respectively) and the control arm comprised 6 boys and 3 girls (percentage 66.7% and 33.3%, respectively).

Inclusion criteria involved children and adolescents (i) with a BMI > 120% of the 95th percentile according to the World Health Organization (WHO), (ii) willing to participate in the study, (iii) not practicing any sports activity, (iv) not on any medication.

Exclusion criteria involved children (i) not willing to participate in the study, (ii) already participating in sports activities, (iii) with severe respiratory diseases (asthma, chronic pulmonary insufficiency) or other underlying chronic pediatric diseases, (iv) intellectual disability, disability, (v) on medication.

### 2.2. Ethical Permission

The study was conducted in accordance with the Declaration of Helsinki and approved by the Ethics Committee of the International Hellenic University (27/25-07-2023) and the scientific committee of the Hippocration Hospital (25771/1-6-2023). Also, signed consent was given by the guardians of the children and adolescents for their participation.

### 2.3. Intervention Characteristics

Children in the intervention arm implemented a 16-week program and were evaluated at baseline and at the end of the intervention.

The collection of information regarding the medical history and demographic data of participating children and adolescents was initially performed through the medical files of each child in the clinic’s archive. In the first meeting, parents and children were thoroughly informed about the study protocol and written consent was given by the children’s guardians. Also, at the same meeting, all children were provided with a brochure and a video detailing the therapeutic exercise protocol.

The intervention included DB exercises along with moderate-intensity aerobic exercises that required the participant to work at 40–60% of the heart rate, could be applied in a real environment, such as at home, and was practically applicable for children with morbid obesity. The therapeutic program lasted for 30 min and was applied daily for 4 months (16 weeks) morning and evening. The physiotherapeutic techniques applied by the children in the intervention group involved deep DB alongside breathing exercises, using the upper limbs, strengthening, balance and muscle coordination exercises, which involved all joints and all muscle groups of the body from different positions (supine, prone, quadrupedal, sitting and standing). All therapeutic exercises were accompanied by deep DB in order to achieve maximum inhalation and exhalation. Additionally, in both the intervention and control arms, participants were instructed to avoid static activities such as sitting at a computer or watching television for long periods of time during their free time. Instead, children were advised to participate in physical activities such as walking, going up and down the stairs instead of using the elevator, and generally incorporating movement into their daily lives. In addition, children in the control group were advised to perform an exercise program of their own liking. More specifically, they were instructed to participate in sports activities 2–3 times a week, with their friends (biking, outdoor games), and to walk for at least half an hour per day during their free time and on weekends. They were also advised to practice DB once daily using a Triflo. Although all these activities included aerobic exercises and DB, it was not possible to assess participant compliance as each child followed an individualized intervention program and not a structured one, as in the intervention arm.

For the purposes of the trial, a Triflo breathing device was provided to each child in both groups for the practice of daily DB. The DB exercise was defined as starting with a calm exhalation for 4″, followed by a deep inhalation for 2″, holding for 4″ and then followed by a strong exhalation. The use of the Triflo, in combination with slow and deep breathing can improve lung function and strengthen the respiratory muscles [26,27].

The Triflo device is easy to use. Good training in the use of the Triflo was provided for each child in the hospital. The choice of the Triflo breathing device was made because it provides visual feedback with the 3 balls that are raised. We opted for the specific DB protocol (4″-2″-4″) so that it would be easily performed by children with morbid obesity. According to the intervention protocol, during the inhalation phase (lasting for 4″), the child tries to lift the 3 balls by inhaling strongly through their mouth, into the mouthpiece of the device and expanding the diaphragm. Then, during the phase of holding the air in their lungs (lasting for 2″), children put an effort in keeping all 3 balls up. Finally, during the exhalation phase (lasting for 4″), children contract the diaphragm and expel the air from the lungs as they watch the balls descend. Some children experienced initial difficulties in performing the DB exercises, as well as on how to use the Triflo to produce DB. After appropriate training from the pediatric physiotherapist and several repetitions, the DB technique was adequately mastered and retained by the participants, since most children considered the exercise as fun. Also, at each follow-up at the hospital, all participants were assessed for correct DB exercise using the Triflo, and re-training was provided to any child who experienced difficulties.

Frequent telephone communication was provided, paired with frequent follow-up visits at the clinic to resolve any questions and assess their compliance with the program, and the results from its implementation. A phone call was made once a week to the child’s parents to check its compliance with the protocol. According to the reports of the children in the intervention group and their parents, all children performed the program once a day, usually in the evening, and twice a week they also performed it in the morning, usually on the weekend when they had no other activities.

### 2.4. Outcomes of Interest

All assessments were carried out at the beginning and end of the intervention and were based on specific indicators. They included anthropometric, cardiorespiratory, hematological, metabolic measurements and psychometric questionnaires completed by the participants and their parents.

#### 2.4.1. Anthropometric Assessment

Anthropometric measurements were performed (height, weight, BMI z-score, arm, waist, hip, thigh and calf circumferences, abdominal, triceps and scapula skinfolds) and the waist-to-height ratio measurement. To calculate the BMI and the degree of obesity in children and adolescents, the WHO definition [28] (expressed as weight in kilograms divided by height in meters squared), and the International criteria by Cole et al. [29] were used. Body weight and stature of participants were measured to the nearest g and cm, respectively, using a Seca 700 mechanical scale (Seca, Hamburg, Germany) and a Harpenden wall—mounted stadiometer (Holtain, Crymych, UK). An anthropometric tape (Cescorf, Porto Alegre, Brazil) was used to measure the circumferences of the waist, hips, arm, thighs, and calves. The measurement of the abdominal, triceps, and scapular skinfolds was performed with the Innovare Skinfold Caliper (Cescorf, Porto Alegre, Brazil).

Central obesity was evaluated by measuring the waist-to-height ratio (WHtR). According to the Overweight and Obesity Management NICE Guideline 2025 [30], most studies agree that the WHtR averages around 0.5. The same cut-off value is suggested for children and adolescents ≥ 6 years of age as for adults for the assessment of intra-abdominal fat. All BMI z-scores (BMIz) were calculated using the Word Health Organization (WHO) Anthro Software v1.0.4 (WHO, Geneva, Switzerland) [31].

#### 2.4.2. Respiratory Fitness and BP

Forced expiratory volume in the first second (FEV1) and forced vital capacity (FVC) parameters were recorded by spirometry, and blood pressure (BP), pulses and oxygen saturation (SpO_2_) were assessed. Spirometry of FVC and FEV1 were evaluated using an electronic volume spirometer, dry type (SENSOR MEDICS, FLOWSENSOR, VMAX SERIES, V.20-1Λ).

For the measurement of BP, the OMRON EVOLV-HEM-7600T-E electronic arm BP monitor was used. For the measurement of SpO_2_ and heart rate, the OMRON SAFE HEART SHO finger oximeter was used. Both tools show high validity and reliability and are widely accepted in the scientific community, the former for the measurement of BP, and the latter for the measurement of SpO_2_ and heart rate.

#### 2.4.3. Blood Tests and Assays

Blood was drawn from each participant following a 12 h fast, for the assessment of complete blood count, glucose concentrations, uric acid, triglycerides, aspartate aminotransferase/serum glutamic-oxaloacetic transaminase, (AST/SGOT), alanine aminotransferase/serum glutamate pyruvate transaminase (ALT/SGPT), high-density lipoprotein (HDL), low-density lipoprotein (LDL), insulin, glycosylated hemoglobin (HbA1c), adrenocorticotropic hormone (ACTH), cortisol, the Homeostatic Model Assessment for Insulin Resistance (HOMA-IR), 17-Hydroxyprogesterone (17-OH), sulfato-dehydroepiandrosterone (S-DHEA), and sex-hormone-binding globulin (SHBG). The assessment of biochemical markers (glucose, SGOT, SGPT, HDL, LDL, uric acid) was performed in blood serum samples on the Beckman Coulter Immunoassay DxI 800 automatic analyzer. The methods used were either enzymatic or chemical. The determination of insulin concentration was also performed on blood serum samples using the Siemens Immulite 2000 XRi immunoassay analyzer. Regarding the HOMA-IR, it was determined with the following formula: HOMA-IR = glucose (mg/dL) × insulin (units/Lt)/405. HbA1c was assessed in whole blood samples with EDTA anticoagulant, on the G7 Standard analyzer.

#### 2.4.4. Psychological Assessments

Participants in both groups were also asked to complete the State-Trait Anxiety Inventory for Children 1/2 (STAIC1/STAIC2) questionnaires, assessing anxiety levels. The STAIC consists of two scales, the STAIC1 and the STAIC2. The STAIC1 is a scale that assesses state anxiety using 20 questions, evaluating the subjective feelings of fear, nervousness and worry. Each question is accompanied by a 3-point response scale and requires the child to indicate the “intensity” of the emotion experienced at the time of the test. Questions 1, 3, 6, 8, 10, 12, 13, 14, 17 and 20 indicate the absence of anxiety and are scored 1, 2 and 3, while questions 2, 4, 5, 7, 9, 11, 15, 16, 18, 19 indicating the presence of anxiety and being scored inversely, i.e., 3, 2, 1. The total value of the scale ranges between 20 and 60 where higher values indicate a higher level of state anxiety. The STAIC1 was successfully applied to the Greek pediatric population and the internal consistency (Cronbach alpha) and reliability (test–retest reliability) indices of the scale in the Greek population were reported to be 0.85 and 0.83, respectively [32].

The STAIC2 is a trait anxiety scale (A-Trait) that includes 20 questions destined to assess individual differences in the way children experience stressful situations in their daily lives. High values on the trait anxiety scale indicate children who perceive social situations as more threatening, as well as children who react with intensity to difficult situations. The trait anxiety scale requires the child to answer each statement by specifying the frequency of occurrence of the behavior described. All statements are indicative of the presence of anxiety and the response options are “very often”, “sometimes”, “rarely” and are scored with 3, 2 and 1, respectively. The total score ranges from 20 to 60. The internal consistency (Cronbach alpha) and reliability (test–retest reliability) of the scale in the Greek population were 0.80 and 0.81, respectively [32].

Self-esteem was evaluated using the Tennessee Self-Concept Scale (TSCS:2) and its six sub-dimensions (physical appearance, moral, personal, family, social and academic self-concept). The TSCS:2 [33,34] assesses children’s self-image and self-esteem. It includes 76 questions with answer options “no”, “maybe”, or “yes” and are scored with 1, 2, 3, respectively. The tool has been previously weighted in the Greek population. The six dimensions evaluated include (a) physical appearance, (b) moral, (c) personal, (d) family, (e) social and (f) academic self-concept. The internal consistency index (Cronbach α) of the scale in the Greek population for childhood was reported to be 0.79, and for adults it ranged between 0.66 and 0.92. The reliability index test–retest reliability ranged between 0.55 and 0.83.

Both the STAIC1/STAIC2 anxiety questionnaires and the TSCS:2 had objective difficulties in their practical application. Some questions were difficult to understand, especially for younger children. In addition, the TSCS:2 scale includes 76 questions and requires more time and concentration for completion. In all these difficulties, support, guidance and assistance were provided to the children by the pediatric physiotherapist and their parents to overcome the difficulties and answer all the questions objectively, before and after the end of the intervention period.

### 2.5. Statistical Analyses

Due to the sample size and mainly due to the unequal distribution of the sample in terms of the two groups, statistical tests were applied with non-parametric methods; specifically, the Mann–Whitney U test was applied to check the differences between the independent variables and the Wilcoxon test was used to investigate differences between the initial and final measurement.

Quantitative variables are presented as median value and interquartile range (25th–75th percentile). In addition, to evaluate the effect of the differences between the means of the two groups or between the measurements, the standardized effect size was used, with the formula r = z/√N. Regarding the psychometric tools for assessing children’s anxiety and self-esteem, reliability and internal consistency were initially applied, with the Cronbach’s alpha index. Due to the size of the sample, there was a thorough reliability check, investigating both the correlation of each question with the entire dimension, but also whether the value of the index differs in case of removing a question from the analysis.

To ensure comparability between the treatment and control arms and minimize possible confounding, we applied propensity score matching. Propensity scores were estimated using the covariates: gender, weight, BMI, and BMIz. The matching itself was conducted using optimal matching without replacement. We assessed the success of the matching, specifically regarding covariate balance, by examining standardized mean differences (SMD) before and after the procedure. Following matching, all covariates showed excellent balance, with SMD values ranging from 0.244 to 0.343.

All statistical tests were performed with the statistical package IBM SPSS Statistics version 27 (IBM, Armonk, NY, USA), while in all statistical tests, the level of statistical significance was set at *p* < 0.05.

## 3. Results

### 3.1. Results of Anthropometric Characteristics and Cardiorespiratory Variables Between Groups, Before and After the Intervention

Table 1 presents the comparisons of anthropometric characteristics and cardiorespiratory variables of the intervention arm at baseline and post intervention. At the end of treatment body weight and BMIz were significantly improved, alongside FEV1, systolic arterial pressure (SAP), SpO_2_, pulse, and all anthropometric indices.

Table 2 presents the comparisons of anthropometric characteristics and cardiorespiratory variables of the control arm at baseline and post intervention. At the end of treatment, body weight, BP, pulse, hips perimeter and abdominal skinfolds were significantly improved.

Between groups (intervention vs. control) comparisons of the anthropometric and cardiorespiratory variables of participants are presented in Table 3. Significant improvements were observed in the intervention arm regarding body weight, BMI z-score, WHtR, all body circumferences, FEV1, BP, pulse and SpO_2_, compared to the control group.

### 3.2. Results of the Hematological and Metabolic Markers of Each Study Arm at Baseline, and Post-Intervention

Table 4 details the hematological and metabolic markers of participants in the intervention group, pre- and post-intervention. In the intervention arm, significant decreases were observed in the uric acid, HDL, ALT/SGPT, insulin, HOMA-IR and SHBG of participants. Improvements in the insulin levels, HOMA-IR and ALT/SGPT were only apparent in the total sample, and among boys.

Table 5 lists the changes in the hematological and metabolic markers of participants in the control arm, pre- and post-intervention. After the intervention, controls did not experience any improvements with regard to their metabolic indices and hematological parameters.

Table 6 details the changes in hematological and metabolic parameters (at end of treatment compared to baseline), between study arms. Uric acid, HDL, AST/SGIT, ALT/SGPT, insulin, HOMA-IR, and SHBG were significantly improved in the intervention group compared to the controls, at 4 months post-treatment.

### 3.3. Psychometric Results

Table 7 lists the psychometric results of participating children in the intervention arm, pre- and post-intervention. All STAIC and TSCS:2 domains were improved at the end of treatment.

Table 8 details the psychometric results of participants in the control arm, pre- and post-intervention. At the end of treatment, general self-perception was reduced in the boys compared to baseline.

Finally, Table 9 details the changes induced in psychometric results post-intervention, between study arms. Regarding the STAIC, state and trait anxiety were improved in the intervention arm. According to the dimensions of the TSCS:2, physical appearance, personal, family, social, academic and self-perception were improved in the intervention arm, compared to the control group.

### 3.4. Restrictions

Statistically significant differences should be read with caution as, based on the calculation of the post hoc power (1-β err), for this specific sample and for an effect size of 0.35 to 0.74, the probability of correctly rejecting the null hypothesis ranges from 12% to 39%. The highest effect size is observed in the anthropometric variables where it is observed that there are indeed substantial changes in the children in the intervention group.

## 4. Discussion

The present study evaluated the effects of a stress management program using DB and physiotherapeutic exercise on the anthropometric, cardiorespiratory, metabolic and psychometric indices of children and adolescents with severe obesity, aged 5–18 years. The main finding of the study was that the intervention was successful in improving body composition, cardiorespiratory fitness, metabolic indices, and psychometric characteristics of the participants in the intervention arm.

In relation to body composition and obesity-related variables, improvements were observed in the children belonging to the intervention group, compared to those in the control arm. In further detail, children in the intervention group experienced decreases in body weight and BMIz, post-intervention. Measurements of the circumferences of the arm, hips, waist and calves were reduced, and improvements were also observed in the skinfolds of the triceps, scapula, abdomen and WHtR. Waist circumference and abdominal skinfold correlate with visceral fat content [35,36] and are more reliable measurements compared to BMI [37]. BMI does not provide information on the distribution of body fat, and more specifically on the content of visceral fat in the body, and for this reason it does not reflect the cardiometabolic health risks of the individual caused by excessive obesity [36,38,39]. Therefore, focusing on WHtR rather than BMI provides a useful indicator of metabolic health [36].

According to a systematic review, the combination of an aerobic exercise program together with resistance exercise improves body composition, inflammation and metabolic markers in children and adolescents with obesity [40], whereas aerobic exercise interventions can improve total body weight, and BMI [41]. In a clinical study conducted in adolescent boys with severe obesity, an aerobics exercise program with maximum heart rate intensity of 60–80% accompanied by resistance exercises led to a reduction in body weight, BMI, and lean body mass by 12.43%, 13.51%, and 5.83%, respectively [42]. In other stress management studies conducted in children and adolescents with obesity, aerobic exercise and DB improved waist-to-hip ratio [22], reduced BMI, anxiety and depression [21]. In children and adolescents with obesity and a BMIz ≥ 2, an annual intervention with recommendations for diet, exercise and lifestyle modification, improves waist circumference or WHtR (indicating abdominal obesity), with ameliorated FEV1/FVC, forced mid-expiratory flow (FEF_25–75%_) and FEF_25–75%_/FVC measures [43].

Among participants in the intervention arm, FEV1, systolic BP, SpO_2_ and heart rate were all improved, while no changes were observed in the FVC and diastolic blood pressure (DAP). Sénéchal et al. showed that an increase in cardiorespiratory fitness is associated with improvements in the BMIz, total fat mass, and liver fat of adolescents with overweight and obesity, at risk of developing type 2 diabetes mellitus [44]. Research has also shown that a regular physical activity program for 6 months reduces BP, and improves arterial function and cardiorespiratory fitness in preadolescent children with obesity [45,46,47]. In similar interventions involving combined aerobic exercise protocols with DB in children and adolescents with obesity, improvements were also noted in the DAP, heart rate, and cardiorespiratory profile, irrespectively of any changes in body composition [21,48,49,50,51].

Related to the lipid and metabolic profile of participants, according to the results, HDL increased in both arms, but the improvement was only significant in the intervention group, while no improvement was observed in the LDL and triglycerides concentrations in either group. On the other hand, a decrease was observed in ALT/SGPT, uric acid, fasting insulin concentrations, HOMA-IR, as well as a small increase in the SHBG levels of participants of the intervention group. It is known that greater blood insulin levels lead to insulin resistance, which in turn leads to weight gain, obesity and low SHBG concentrations [52]. Therefore, the improvement in HOMA-IR and the small increase in SHBG levels in the intervention group, indicates improved insulin sensitivity. No changes were observed in the HbA1c, cortisol, 17-OH, ACTH, or S-DHEA concentrations. Although participants in the intervention group applied the DB and exercise therapeutic protocol once daily, the non-strict adherence to the protocol twice a day for the entire week, probably led to a smaller and not fully objective result in the levels of improvement in the main stress hormones (cortisol, ACTH, and 17-OH), where no changes were observed at the end of the intervention. Also, the fact that there are no changes in stress hormones including cortisol, ACTH, and 17-OH is likely because blood samples were taken in the hospital and not at the patient’s home before the children getting out of bed, which would have produced more objective results.

Results from previous studies are consistent with the present findings. Evidence indicates that weight loss has been associated with improvements in the prevalence and severity of several obesity-related comorbidities, such as insulin resistance, inflammation, dyslipidemia, hypertension, metabolic syndrome, diabetes, lung and cardiovascular disease [53]. Aerobic exercise, as well as resistance exercise, reduce HOMA-IR by lowering body fat content [45,54]. According to a systematic review, aerobic exercise interventions improve fasting insulin levels, insulin resistance [46,47], blood lipid levels [55], LDL, leptin and cortisol levels [41], and blood visfatin [56] levels in children and adolescents with overweight and obesity.

Although a significant reduction in the HOMA-IR was observed in the intervention arm –indicating the positive effect of exercise and DB–, the final mean group values still indicate the presence of insulin resistance. The effect of exercise on glycemic control in children and adolescents with obesity is not entirely clear. It is important to note that previous studies have shown that exercise interventions do not always lead to statistically significant improvements in HOMA-IR and HDL, LDL, triglycerides, total cholesterol concentrations and insulin resistance in children and adolescents with obesity [57,58]; however, when aerobic exercise is implemented, it is more likely to improve fasting insulin levels, insulin resistance, as well as, blood lipid levels [46,59]. On a side note, resistance exercise alone is not associated with reductions in fat mass, or improvements in metabolic parameters and cardiovascular disease risk factors in children and adolescents with obesity [46]. Studies have shown that a combination of resistance and aerobic exercise is of particular interest, as it provides greater benefits than aerobic exercise or resistance exercise alone, in adolescents with obesity, by improving fasting glucose, insulin, and insulin resistance levels [60,61,62]. Nevertheless, increasing physical activity in the pediatric population with obesity is of paramount importance, as sedentary lifestyle and physical inactivity have been recognized as important factors leading to the development of obesity and metabolic syndrome [57,63,64].

Stress hormones, including cortisol and ACTH failed to improve in the intervention arm, but the observed improvement in BMIz and WHtR could be attributed to a possible reduced activity of the hypothalamic–pituitary–adrenal axis (HPA) axis, which shows increased activity in obesity. DB exercise regulates the HPA function and the action of cortisol on metabolism (liver, muscle, adipose tissue) and can lead to weight loss. This could be the direct result of stress management on weight loss. The stress system interacts with various homeostatic systems and neurobiochemical mediators, such as serotonin and corticotropin, thus, it is possible that stress management interventions reduce anxiety and depression, leading to weight loss indirectly, by affecting the expression of these hormones. The diaphragm is innervated by the phrenic nerve which is responsible for its function. The phrenic nerve in its course is connected to the vagus nerve. Systematic and deep DB exercises through intra-abdominal pressure and the phrenic nerve, enhance the tone of the vagus, an action that can affect whole body function, bring relaxation and balance [65].

Regarding the comparison of the two groups in terms of psychological indicators, an improvement was noted in the intervention arm compared to the control, in terms of temporary anxiety, the dimension of “general self-perception”, as well as the dimensions “personal”, “family” and “social”. In a similar clinical study in adolescent boys with severe stress obesity, an aerobic exercise program combined with resistance training reduced oxidative and inducing an increase in antioxidant enzymes [42,66]. Results from 761 studies indicate that DB reduces perceived stress [25]. Stress management programs including DB, physical exercise, muscle relaxation and behavior change training, result in reduced depression, anxiety and introversion, and improved daily habits and school performance, in children and adolescents with obesity [21].

Although the number of children and adolescents with obesity is increasing rapidly, it is still unclear whether psychological problems are associated with obesity as reviews to date, report modest associations between obesity and global self-perception. Griffiths and colleagues [67] revealed that youngsters with obesity are at increased risk of low self-esteem and reduced quality of life. Although it has not yet been confirmed that the improvement in self-esteem and quality of life consists of the epiphenomenon of weight loss, improvements have been reported in the general self-perception and quality of life in children and adolescents with obesity, when weight loss is achieved [67]. The need to clarify the mechanisms that link obesity with self-perception, psychosocial development, mental health and quality of life in children and adolescents [67,68] is yet to be demonstrated.

Toxic stress negatively affects learning, behavior, and physiological function, leading to chronic diseases and unhealthy lifestyles [69]. Although it has been shown that regular and moderate physical activity reduces anxiety, intense and prolonged exercise can increase physical and psychological stress. Both types of stress activate the neuroendocrine axis and lead to cortisol production, in response to prolonged exercise [70,71]. For this, it is believed that the results of stress management interventions affect anthropometric, metabolic, and psychometric indicators through behavioral and neuroendocrine mechanisms [72,73,74]. In a state of stress, the HPA is activated, which controls the body’s homeostasis through the ANS. Disruption of the homeostasis over a long period of time due to stress, leads to metabolic imbalance, insulin resistance, hypercortisolemia –as cortisol is the main stress hormone–, and chronic low-grade inflammation. On the other hand, DB is related to metabolic balance [66,75]. DB exercises activate the parasympathetic system, leading to relaxation and calmness [23,24], metabolic balance, balance of the ANS and neuroendocrine system, regulating HPA function, while improving insulin resistance, hypercortisolemia and low-grade inflammation.

Childhood obesity leads to metabolic, cardiovascular and psychiatric diseases that follow the individual into adulthood, while increasing the risk of premature mortality. For this reason, early intervention using exercise is crucial to combat comorbidities related to obesity. Most of the available research agrees that the most effective way to achieve [66] significant weight loss and improve lipid profiles is through a combination of aerobic exercise, DB, diet and lifestyle changes. The latest guidelines recommend engaging in at least 150 min of moderate-intensity, or 75 min of vigorous-intensity aerobic exercise per week, including muscle-strengthening exercise—involving all major muscle groups–, performed at least twice weekly [49]. Once weight loss is achieved, maintaining weight loss requires 225–420 min of moderate-intensity exercise on a weekly basis [53,76].

Although the best exercise prescription for children and adolescents with obesity has not been found to date, studies show that the combination of aerobic and muscle-strengthening exercises are promising in the fight against childhood obesity [57].

### Limitations of the Present Research

Compliance with the implementation of the stress management protocol was based on subjective self-reports of the children in the intervention arm. For this reason, there was frequent telephone communication and follow-up meetings at the clinic, involving both the children and their parents, aiming to resolve queries and evaluate protocol compliance.

A weakness of the research herein involves the anthropometric measurements, which, although taken before and after the intervention by the same person, using the same measurement tools, cannot be completely objective, leaving a small standard deviation error in each measurement.

It is important to mention that the 4-month (16-week) intervention period is a reasonable time for investigating the effects of the intervention on the psychometric biomarkers of the individuals in the intervention group, but also quite restrictive.

Another important limitation of the study is that combining aerobic exercise with DB makes it difficult to determine the specific, isolated contribution and effectiveness of DBcompared to aerobic exercise alone.

Last, but not least, the lack of follow-up data after the end of the intervention constitutes an additional limitation of the study.

## 5. Conclusions

In conclusion, in children and adolescents with morbid obesity, daily physiotherapeutic exercise with DB and the use of Triflo seems to have a positive effect on metabolic markers, reducing the risk of low-grade inflammation that leads to long-term morbidity and complications. It also improves their general self-esteem, cardiorespiratory function and mental state, increasing their ability to exercise, including improving personal, family and social relationships and quality of life. For this reason, physiotherapeutic aerobic exercise and DB training should be systematically integrated into the education of all children and adolescents with obesity, so that it becomes their way of life [49,50,51]. It is crucial for children to continue this exercise program twice daily over the long term, so that it becomes a sustainable habit carried into adulthood. The program is enjoyable, simple, effective, and, most importantly, can be performed at home. Future studies should incorporate longer follow-up periods—at least 12 months after the intervention—to evaluate its lasting impact, ideally including a less intensive maintenance phase.

Unfortunately, the prevalence of childhood obesity does not appear to halt [77]. It is important that similar physiotherapy protocols for managing anxiety, stress and depression are recommended by health professionals (doctors, physiotherapists) and implemented by all children and adolescents, especially those with overweight and obesity, in order to improve anxiety, stress and depression.

A simple, practical guide that brings together all these approaches could be developed and incorporated into international guidelines for preventing and combating childhood obesity. This guide could help inform children, adolescents, and their parents—through parent associations, as well as the educational and medical communities—about the severe impact of obesity on childhood, adolescent, and adult health, its economic burden due to related health conditions, and, most importantly, the urgent need for effective prevention strategies. The guide could include an easy-to-follow protocol featuring moderate-intensity aerobic activities combined with modern deep DB exercises and the use of the Triflo breathing device, making the program more enjoyable and effective for children and adolescents aged 5–18, regardless of whether they are of normal weight or obese. The program should also be adaptable to individual needs. It is vital to highlight the importance of daily DB exercises, either alone or alongside low-intensity aerobic activities, for children’s physical and mental well-being. Schools offer a safe and ideal setting for this guide to be integrated into school curricula as part of physical education and skill-building activities. This approach would ensure that all children, regardless of weight, participate equally, helping prevent obesity and fostering healthy habits that can improve their overall quality of life.

## Figures and Tables

**Table 1:** Anthropometric characteristics and cardiorespiratory fitness pre- and post-intervention, in the intervention arm (N = 22) ^‡^.

	Baseline			EOT		
Variables	Total (*n* = 22)	Boys (*n* = 12)	Girls (*n* = 10)	Total (*n* = 22)	Boys (*n* = 12)	Girls (*n* = 10)
Body weight (kg)	80.2 ± 18.3	78.5 ± 16.7	82.3 ± 20.7	77.2 ± 17.6 ***	76.1 ± 16.9 *	78.5 ± 19.3 *
BMIz	3.6 ± 0.8	3.5 ± 0.8	3.7 ± 0.8	3.3 ± 0.7 ***	3.2 ± 0.7 **	3.3 ± 0.8 **
FVC (%)	3.3 ± 0.9	3.2 ± 0.8	3.5 ± 1.1	3.3 ± 0.8	3.4 ± 0.9	3.2 ± 0.7
FEV1 (%)	2.7 ± 0.6	2.7 ± 0.7	2.6 ± 0.5	2.8 ± 0.6 *	2.8 ± 0.7	2.7 ± 0.5
SAP (mmHg)	123.8 ± 14.5	123.7 ± 14.8	123.9 ± 14.9	119.2 ± 10.6 *	119 ± 11.7	119.4 ± 9.8
DAP (mmHg)	71.6 ± 10.9	74.3 ± 13.3	68.3 ± 6.1	71.8 ± 8.6	72.7 ± 10.2	70.8 ± 6.5
Pulse (bpm)	90.9 ± 15.3	92.9 ± 17.8	88.5 ± 12.2	84 ± 11.7 **	85.9 ± 12.9 *	81.7 ± 10.4
SpO_2_ (%)	97.7 ± 1.4	97.4 ± 1.8	98 ± 0.7	98.6 ± 1 ***	98.3 ± 1.2 *	98.8 ± 0.8 **
Arm circumference (cm)	33.8 ± 4.3	32.2 ± 3.6	35.8 ± 4.5	32.2 ± 3.6 ***	31.2 ± 3.5 *	33.5 ± 3.4 **
Waist perimeter (cm)	94.3 ± 11.8	95 ± 7.8	93.5 ± 15.8	89.9 ± 10.5 ***	90.8 ± 8.1 **	88.9 ± 13.2 **
Hips perimeter (cm)	110.3 ± 12	108.3 ± 9.2	112.8 ± 14.9	106.5 ± 11.6 ***	103.9 ± 9.4 **	109.7 ± 13.7 **
Thigh perimeter (cm)	58.7 ± 7.6	55.8 ± 7	62.1 ± 7.1	56.3 ± 7.5 ***	53.3 ± 6.7 **	59.9 ± 7.0 **
Calf perimeter (cm)	40.8 ± 4.2	39.5 ± 4.3	42 ± 3.9	39.7 ± 4.2 ***	38.6 ± 4.4 **	40.7 ± 3.8 **
Triceps skinfold (cm)	28 ± 6.9	28.4 ± 6.8	27.4 ± 7.4	25.9 ± 6.5 ***	26.2 ± 6.3 **	25.5 ± 7.1 **
Scapular skinfold (cm)	31.7 ± 8.5	33.9 ± 8.3	29.1 ± 8.3	29.7 ± 7.5 ***	31.5 ± 6.8 **	27.5 ± 8.1 **
Abdominal skinfold (cm)	40.2 ± 10.7	39.8 ± 7	40.8 ± 14.4	36.6 ± 9.4 ***	36.3 ± 6 **	37 ± 12.8 **
WHtR	0.62 ± 0.07	0.63 ± 0.05	0.61 ± 0.09	0.58 ± 0.06 ***	0.58 ± 0.04 **	0.57 ± 0.07 **

BMIz, body mass index z-score; DAP, diastolic arterial pressure; FVC, forced vital capacity; FEV1, forced expiratory volume in the first second; SAP, systolic arterial pressure; SD, standard deviation; SpO_2_, oxygen saturation; WHtR, waist-to-height ratio; ^‡^ Data are presented as means ± SD; statistically significant compared to baseline: *** *p* ≤ 0.001; ** *p* ≤ 0.01; * *p* ≤ 0.05.

**Table 2:** Anthropometric characteristics and cardiorespiratory fitness pre- and post-intervention, in the control group (N = 9) ^‡^.

Variables	Baseline			EOT		
	Total (*n* = 9)	Boys (*n* = 6)	Girls (*n* = 3)	Total (*n* = 9)	Boys (*n* = 6)	Girls (*n* = 3)
Body weight (kg)	94.4 ± 24.7	89.9 ± 31.4	102.1 ± 5.6	95.5 ± 24.5 *	91.1 ± 31.3 *	102.8 ± 4.3
BMIz	4.02 ± 0.81	3.93 ± 0.61	4.14 ± 1.18	3.97 ± 0.76	3.93 ± 0.63	4.02 ± 1.06
FVC (%)	3.8 ± 1.1	3.9 ± 1.3	3.6 ± 0.6	3.8 ± 1.1	3.9 ± 1.3	3.6 ± 0.7
FEV1 (%)	3.1 ± 0.8	3.2 ± 1	3 ± 0.1	3.1 ± 0.8	3.2 ± 1	2.9 ± 0.1
SAP (mmHg)	114.6 ± 12.8	118.7 ± 11.7	106.3 ± 12.5	117.6 ± 12.3 *	121.7 ± 10.7 *	109.3 ± 12.9
DAP (mmHg)	69.4 ± 9.5	68 ± 10.7	72.3 ± 7.5	75 ± 10 **	74.2 ± 10.2 *	76.7 ± 11.5
Pulse (bpm)	88.2 ± 16.5	92 ± 14.6	80.7 ± 20.6	91 ± 15.9 *	94.5 ± 14.6	84 ± 19.1
SpO_2_ (%)	97.9 ± 0.6	98 ± 0.6	97.7 ± 0.6	98 ± 0.5	98 ± 0.6	98 ± 0
Arm circumference (cm)	39.8 ± 7.2	39.8 ± 9.1	40 ± 1	40.1 ± 7.2	40 ± 9.1	40.3 ± 1.5
Waist perimeter (cm)	106.6 ± 16.6	108 ± 20.7	103.7 ± 2.9	107.5 ± 15.5	108.2 ± 19.5	106.2 ± 2.9
Hips perimeter (cm)	124.9 ± 19.7	123.1 ± 24.6	128.5 ± 2.6	125.9 ± 19.1 *	123.7 ± 23.7	130.3 ± 3.3
Thigh perimeter (cm)	72.6 ± 14.2	72.1 ± 17	73.7 ± 9.3	72.9 ± 15.1	72.8 ± 18.2	73.3 ± 9.1
Calf perimeter (cm)	46.5 ± 4.8	46.3 ± 5.8	46.8 ± 3	46.6 ± 4.5	46.2 ± 5.4	47.5 ± 2.8
Triceps skinfold (cm)	23.8 ± 6.4	23.7 ± 6.3	24 ± 7.9	24.2 ± 6.6	23.5 ± 6.3	25.7 ± 8.5
Scapular skinfold (cm)	26.4 ± 11.5	22.3 ± 9	34.7 ± 13.1	26.9 ± 10.8	22.9 ± 8.7	34.9 ± 11.4
Abdominal skinfold (cm)	41.4 ± 12.7	40.2 ± 13.9	44 ± 12.2	42.2 ± 12.6 *	41 ± 13.6	44.7 ± 12.4
WHtR	0.68 ± 0.07	0.68 ± 0.08	0.67 ± 0.07	0.67 ± 0.07	0.68 ± 0.08	0.63 ± 0.06

BMIz, body mass index z-score; DAP, diastolic arterial pressure; FVC, forced vital capacity; FEV1, forced expiratory volume in the first second; SAP, systolic arterial pressure; SD, standard deviation; SpO_2_, oxygen saturation; WHtR, waist-to-height ratio; ^‡^ Data are presented as means ± SD; statistically significant compared to baseline: ** *p* ≤ 0.01; * *p* ≤ 0.05.

**Table 3:** Comparisons of change (Δ) in anthropometric characteristics and cardiorespiratory variables at baseline and post intervention, between groups.

	Intervention (*n* = 22)	Control (*n* = 9)	Mean Difference (SED)	95% CI
Variables	Δ (Mean ± SD)	Δ (Mean ± SD)		
Body weight (kg)	−3.0 ± 4.6	1.1 ± 1.0 ^†††^	−4.1 (1.7)	(−7.5 to −0.7)
BMIz	−0.4 ± 0.2	−0.1 ± 0.1 ^†††^	−0.3 (0.1)	(−0.5 to −0.1)
FVC (%)	0.0 ± 0.5	0.0 ± 0.1	0 (0.2)	(−0.4 to 0.3)
FEV1 (%)	0.1 ± 0.2	0.0 ± 0.0 ^†^	0.1 (0.1)	(0 to 0.2)
SAP (mmHg)	−4.6 ± 10.3	3.0 ± 1.7 ^††^	−7.6 (3.5)	(−14.7 to −0.5)
DAP (mmHg)	0.3 ± 7.5	5.6 ± 2.7 ^†^	−5.3 (2.6)	(−10.6 to 0)
Pulse (bpm)	−6.9 ± 9.2	2.8 ± 2.3 ^††^	−9.7 (3.1)	(−16.1 to −3.3)
SpO_2_ (%)	0.9 ± 0.8	0.1 ± 0.3 ^†^	0.8 (0.3)	(0.2 to 1.3)
Arm circumference (cm)	−1.6 ± 1.7	0.3 ± 0.4 ^††^	−1.9 (0.6)	(−3.1 to −0.7)
Waist perimeter (cm)	−4.4 ± 3.9	1.0 ± 1.7 ^†††^	−5.4 (1.4)	(−8.1 to −2.6)
Hips perimeter (cm)	−3.8 ± 2.1	1.0 ± 1.2 ^†††^	−4.8 (0.8)	(−6.3 to −3.2)
Thigh perimeter (cm)	−2.4 ± 1.7	0.3 ± 1.3 ^†††^	−2.7 (0.6)	(−4 to −1.4)
Calf perimeter (cm)	−1.1 ± 0.6	0.1 ± 0.9 ^†††^	−1.2 (0.3)	(−1.8 to −0.7)
Triceps skinfold (cm)	−2.1 ± 1.8	0.4 ± 1.4 ^†††^	−2.5 (0.7)	(−3.9 to −1.1)
Scapular skinfold (cm)	−2.1 ± 1.9	0.5 ± 1.3 ^†††^	−2.5 (0.7)	(−3.9 to −1.1)
Abdominal skinfold (cm)	−3.6 ± 2.3	0.8 ± 0.7 ^†††^	−4.4 (0.8)	(−6 to −2.8)
WHtR	−0.04 ± 0.04	−0.01 ± 0.03 ^†^	0.0 (0.0)	(−0.1 to 0)

BMIz, body mass index z-score; CI, confidence intervals; DAP, diastolic arterial pressure; FVC, forced vital capacity; FEV1, forced expiratory volume in the first second; SAP, systolic arterial pressure; SD, standard deviation; SED, standard error of difference; SpO_2_, oxygen saturation; WHtR, waist-to-height ratio; statistically significant compared to intervention: ^†††^ *p* ≤ 0.001; ^††^ *p* ≤ 0.01; ^†^ *p* ≤ 0.05.

**Table 4:** Hematological and metabolic markers of participants in the intervention group, pre- and post-intervention (N = 22) ^‡^.

		Baseline			EOT	
Variables	Total (*n* = 22)	Boys (*n* = 12)	Girls (*n* = 10)	Total (*n* = 22)	Boys (*n* = 12)	Girls (*n* = 10)
Glucose (mg/dL)	92.0 ± 15.25	94.3 ± 13	89.2 ± 17.9	90.5 ± 9.7	93.1 ± 8.8	87.3 ± 10.2
Uric acid (mg/dL)	5.7 ± 1.1	5.6 ± 1	5.8 ± 1.1	5.2 ± 1.1 ***	5.1 ± 1 **	5.3 ± 1.3 *
Triglycerides (mg/dL)	85.8 ± 27.5	84.9 ± 29.4	87 ± 26.3	91.8 ± 34.1	90.3 ± 30.4	93.7 ± 40.3
HDL (mg/dL)	44.2 ± 7.6	45.3 ± 8.8	42.9 ± 6.2	49.3 ± 8.8 ***	49.5 ± 11.3 **	49.1 ± 5 **
LDL (mg/dL)	96.8 ± 25.2	103.4 ± 28.4	87.9 ± 18.1	96.4 ± 24.1	99.1 ± 28.5	92.8 ± 17.8
(AST/SGOT) (U/L)	26.1 ± 9.2	24.9 ± 9	27.6 ± 9.75	22.4 ± 8.3	24.4 ± 10	20 ± 4.99
(ALT/SGPT) (U/L)	31.2 ± 24.3	28.1 ± 14.8	34.9 ± 32.8	21.2 ± 8.9 **	21.8 ± 10.2 *	20.6 ± 7.7
HbA1c (%)	5.2 ± 0.3	5.2 ± 0.3	5.1 ± 0.3	5.2 ± 0.3	5.3 ± 0.4	5.1 ± 0.1
Insulin (mlU/L)	23.5 ± 42.2	16.4 ± 14.6	32.1 ± 61.2	10.4 ± 6.7 **	8.4 ± 3.2 **	12.8 ± 9
Cortisol (μg/dL)	9.3 ± 4	8.8 ± 4	10.1 ± 4.1	8.5 ± 2.5	8 ± 1.9	9.3 ± 3.3
ACTH (pg/mL)	24 ± 16.1	20.4 ± 10.7	28.3 ± 20.7	28.4 ± 19.5	22.9 ± 9.7	35.1 ± 26.1
HOMA-IR	6.5 ± 14.4	4.2 ± 4.7	9.3 ± 21	2.3 ± 1.5 ***	1.9 ± 0.8 **	2.7 ± 2
17-OH (ng/mL)	0.8 ± 0.4	0.7 ± 0.3	0.9 ± 0.5	0.8 ± 0.4	0.6 ± 0.3	0.9 ± 0.5
S-DHEA (μg/100 mL)	175.5 ± 119.7	174 ± 141	177.3 ± 95.5	188.8 ± 102.6	177.7 ± 118.7	202.2 ± 83.5
SHBG (nmol/L)	19.9 ± 8.6	18.3 ± 8.7	21.8 ± 8.5	26 ± 11.2 ***	25.3 ± 13.2 **	26.8 ± 8.8 **

17-OH, Hydroxyprogesterone; ACTH, adreocorticotropic hormone; ALT, alanine aminotransferase; AST, aspartate aminotransferase; EOT, end of treatment; HbA1c, glycosylated hemoglobin; HDL, high-density lipoprotein; HOMA-IR, Homeostatic Model Assessment for Insulin Resistance; LDL, low-density lipoprotein; SD, standard deviation; S-DHEA, sulfato–dehydroepiandrosterone; SGOT, serum glutamic-oxaloacetic transaminase; SGPT, serum glutamate pyruvate transaminase; SHBG, sex-hormone-binding globulin; ^‡^ Data are presented as means ± SD; statistically significant compared to baseline: *** *p* ≤ 0.001; ** *p* ≤ 0.01; * *p* ≤ 0.05.

**Table 5:** Hematological and metabolic markers of participants in the control group, pre- and post-intervention (N = 9) ^‡^.

Variables	Baseline			EOT		
	Total (*n* = 9)	Boys (*n* = 6)	Girls (*n* = 3)	Total (*n* = 9)	Boys (*n* = 6)	Girls (*n* = 3)
Glucose (mg/dL)	83.9 ± 8	86.7 ± 7.3	78.3 ± 7.4	82.7 ± 8.3	84.3 ± 7.7	79.3 ± 10
Uric acid (mg/dL)	6.4 ± 1.7	6.6 ± 1.9	5.7 ± 0.1	6.9 ± 1.6	7.4 ± 1.5	5.5 ± 0.2
Triglycerides (mg/dL)	103 ± 41.5	114.7 ± 41.7	68 ± 9.9	103.4 ± 41.3	114.7 ± 41.9	69.5 ± 9.2
HDL (mg/dL)	41.1 ± 6.7	43.8 ± 5	33 ± 4.2	41.4 ± 7.8	44.7 ± 5.5	31.5 ± 3.5
LDL (mg/dL)	99.9 ± 22.7	105.7 ± 22.7	82.5 ± 14.8	100.1 ± 22.3	105.7 ± 22.5	83.5 ± 14.8
(AST/SGOT) (U/L)	21.8 ± 6.9	25.2 ± 5.8	15 ± 1.7	22.1 ± 6.6	25.8 ± 4.5	14.8 ± 2
(ALT/SGPT) (U/L)	32 ± 24.8	39.3 ± 28.1	17.3 ± 3.1	32.4 ± 25.2	39.9 ± 28.5	17.3 ± 3.2
HbA1c (%)	5.4 ± 0.4	5.4 ± 0.4	5.4 ± 0.4	5.5 ± 0.3	5.5 ± 0.4	5.5 ± 0.3
Insulin (mlU/L)	17 ± 9.6	19.9 ± 10.6	11.1 ± 3.2	17.6 ± 10.3	21.11 ± 11.1	10.59 ± 2.6
Cortisol (μg/dL)	8.8 ± 3.8	8.3 ± 4.1	9.7 ± 4.1	8.7 ± 3.3	8 ± 3.7	10.1 ± 3
ACTH (pg/mL)	19.8 ± 22.9	20.3 ± 27.6	18.9 ± 13.4	20.3 ± 21.7	21.6 ± 26.4	17.6 ± 11.4
HOMA-IR	3.6 ± 2.4	4.4 ± 2.7	2.13 ± 0.59	3.7 ± 2.6	4.6 ± 2.8	2.05 ± 0.47
17-OH (ng/mL)	0.7 ± 0.3	0.6 ± 0.1	1 ± 0.4	0.8 ± 0.5	0.6 ± 0.4	1.3 ± 0.4
S-DHEA (μg/100 mL)	168.9 ± 88.9	177.5 ± 108.5	151.7 ± 39.1	172.9 ± 97.7	180.2 ± 118.7	158.4 ± 50.2
SHBG (nmol/L)	18.0 ± 2.7	17.6 ± 3	18.7 ± 2.3	18.1 ± 3.5	16.9 ± 3.7	20.4 ± 1.6

17-OH, Hydroxyprogesterone; ACTH, adreocorticotropic hormone; ALT, alanine aminotransferase; AST, aspartate aminotransferase; EOT, end of treatment; HbA1c, glycosylated hemoglobin; HDL, high-density lipoprotein; HOMA-IR, Homeostatic Model Assessment for Insulin Resistance; LDL, low-density lipoprotein; SD, standard deviation; S-DHEA, sulfato–dehydroepiandrosterone; SGOT, serum glutamic-oxaloacetic transaminase; SGPT, serum glutamate pyruvate transaminase; SHBG, sex-hormone-binding globulin; ^‡^ Data are presented as means ± SD.

**Table 6:** Comparisons of change (Δ) in the hematological and metabolic markers at baseline and post-intervention, between groups.

	Intervention (*n* = 22)	Control (*n* = 9)	Mean Difference (SED)	95% CI
Variables	Δ (mean ± SD)	Δ (mean ± SD)		
Glucose (mg/dL)	−1.5 ± 15.7	−1.2 ± 2.8	−0.3 (5.3)	(−11.2 to 10.6)
Uric acid (mg/dL)	−0.6 ± 0.6	0.6 ± 1.2 ^††^	−1.1 (0.3)	(−1.8 to −0.4)
Triglycerides (mg/dL)	6.0 ± 22.9	0.4 ± 2.3	5.6 (8.2)	(−11.3 to 22.4)
HDL (mg/dL)	5.1 ± 3.8	0.3 ± 1.5 ^†††^	4.9 (1.4)	(2 to 7.7)
LDL (mg/dL)	−0.4 ± 13.3	0.3 ± 1.4	−0.6 (4.8)	(−10.4 to 9.2)
(AST/SGOT) (U/L)	−3.7 ± 10.6	0.3 ± 1.5 ^†^	−4.1 (3.6)	(−11.4 to 3.3)
(ALT/SGPT) (U/L)	−10.0 ± 20.1	0.4 ± 1.5 ^†^	−10.3 (6.8)	(−24.2 to 3.5)
HbA1c (%)	0.0 ± 0.3	0.1 ± 0.1	0.0 (0.1)	(−0.3 to 0.2)
Insulin (mlU/L)	−13.2 ± 37.9	0.6 ± 1.2 ^†††^	−13.8 (12.8)	(−39.9 to 12.3)
Cortisol (μg/dL)	−0.8 ± 3.4	−0.1 ± 0.8	−0.7 (1.4)	(−3.7 to 2.3)
ACTH (pg/mL)	4.4 ± 18.8	0.5 ± 2.2	4 (6.3)	(−9 to 16.9)
HOMA-IR	−4.2 ± 13.3	0.1 ± 0.3 ^†††^	−4.3 (4.5)	(−13.5 to 4.9)
17-OH (ng/mL)	0.0 ± 0.3	0.1 ± 0.3	−0.1 (0.1)	(−0.4 to 0.2)
S-DHEA (μg/100 mL)	13.4 ± 89.4	4.0 ± 31.5	9.4 (30.8)	(−53.7 to 72.4)
SHBG (nmol/L)	6.2 ± 5.1	0.1 ± 2.0 ^†††^	6.1 (1.8)	(2.4 to 9.7)

17-OH, Hydroxyprogesterone; ACTH, adreocorticotropic hormone; ALT, alanine aminotransferase; AST, aspartate aminotransferase; CI, confidence intervals; HbA1c, glycosylated hemoglobin; HDL, high-density lipoprotein; HOMA-IR, Homeostatic Model Assessment for Insulin Resistance; LDL, low-density lipoprotein; SD, standard deviation; S-DHEA, sulfato–dehydroepiandrosterone; SED, standard error of difference; SGOT, serum glutamic-oxaloacetic transaminase; SGPT, serum glutamate pyruvate transaminase; SHBG, sex-hormone-binding globulin; statistically significant compared to intervention: ^†††^ *p* ≤ 0.001; ^††^ *p* ≤ 0.01; ^†^ *p* ≤ 0.05.

**Table 7:** Psychometric results of participants in the intervention group, pre- and post-intervention (N = 22) ^‡^.

			Baseline			EOT	
		Total (*n* = 22)	Boys (*n* = 12)	Girls (*n* = 10)	Total (*n* = 22)	Boys (*n* = 12)	Girls (*n* = 10)
STAIC	Absence of stress	19.0 ± 2.6	19.8 ± 2.9	18.1 ± 2	16.4 ± 2.6 ***	17.1 ± 2.3 *	15.6 ± 2.8 *
	Presence of stress	16.4 ± 2.6	16.3 ± 2.7	16.5 ± 2.5	14.0 ± 3.24 **	14.5 ± 3.5	13.4 ± 2.9 *
	State anxiety	35.4 ± 3.5	36.1 ± 3.9	34.6 ± 2.8	30.4 ± 4.3 ***	31.6 ± 4.1 **	29 ± 4.2 **
	Trait anxiety	42.1 ± 3.2	41.8 ± 3.7	42.3 ± 2.6	38.4 ± 7.9 **	38 ± 7.9 *	38.8 ± 8.3
TSCS:2	Physical appearance	21.1 ± 1.9	20.5 ± 2.1	21.8 ± 1.2	25.2 ± 2.7 ***	25.2 ± 3.1 **	25.2 ± 2.3 **
	Moral	19.9 ± 1.8	19.8 ± 2.1	20 ± 1.6	23.1 ± 3.5 **	22.2 ± 3	24.2 ± 3.8 *
	Personal	22.9 ± 2.1	22.4 ± 2	23.4 ± 2.07	26.6 ± 2.6 ***	26.5 ± 2.7 *	26.7 ± 2.67 *
	Family	24.2 ± 1.8	23.5 ± 1.3	25.1 ± 1.9	27.8 ± 3.3 ***	27.5 ± 3.9 **	28.1 ± 2.6 *
	Social	29.8 ± 1.9	29.4 ± 2.1	30.3 ± 1.6	35.1 ± 4.0 ***	36.1 ± 4.3 **	34 ± 3.5 *
	Academic	20.3 ± 2.6	19.9 ± 2.7	20.7 ± 2.45	22.9 ± 3.8 **	22.3 ± 4.1 *	23.6 ± 3.3 *
	General self-perception	138.1 ± 6.4	135.5 ± 6.4	141.3 ± 5	160.6 ± 14.2 ***	159.7 ± 14.7 **	161.8 ± 14.4 **

EOT, end of treatment; STAIC, State-Trait Anxiety Inventory for Children; SD, standard deviation; TSCS:2, Tennessee Self-Concept Scale:2; ^‡^ Data are presented as means ± SD; statistically significant compared to baseline: *** *p* ≤ 0.001; ** *p* ≤ 0.01; * *p* ≤ 0.05.

**Table 8:** Psychometric results of participants of control group, pre- and post-intervention (N = 9) ^‡^.

Variables		Baseline			EOT		
		Total (*n* = 9)	Boys (*n* = 6)	Girls (*n* = 3)	Total (*n* = 9)	Boys (*n* = 6)	Girls (*n* = 3)
STAIC	Absence of stress	20.8 ± 5.2	19.8 ± 5.9	22.7 ± 3.5	20.0 ± 4.4	18.3 ± 4.4	23.3 ± 2.5
	Presence of stress	15.1 ± 2.5	14.3 ± 2	16.7 ± 3.1	15.2 ± 3.0	14.8 ± 2.9	16 ± 3.6
	State anxiety	35.9 ± 6.1	34.2 ± 6	39.3 ± 5.9	35.2 ± 5.8	33.2 ± 4.9	39.3 ± 6.1
	Trait anxiety	42.7 ± 6.4	43.5 ± 5.9	41 ± 8.5	41.9 ±7.0	42.3 ± 8.1	41 ± 5.6
TSCS:2	Physical appearance	22.4 ± 3.3	23.2 ± 3.4	21 ± 3	23.3 ± 3.5	23.2 ± 4	23.7 ± 3.2
	Moral	20.3 ± 2.5	20.5 ± 2.3	20.0 ± 3.5	21.1 ± 2.3	20.7 ± 2.2	22 ± 2.65
	Personal	22.1 ± 3.6	22.5 ± 4	21.3 ± 3.1	21.6 ± 2.9	22 ± 3.3	20.7 ± 2.1
	Family	24.6 ± 2.9	24.7 ± 3.7	24.3 ± 0.6	23.9 ± 2.7	24 ± 3.3	23.7 ± 0.6
	Social	27.2 ± 3.8	27.5 ± 4.7	26.7 ± 1.5	26.0 ± 4.0	26 ± 5	26 ± 1.7
	Academic	21.1 ± 5.9	21.3 ± 4.7	20.7 ± 9.1	19.9 ± 4.6	20.3 ± 4.6	19 ± 5.6
	General self-perception	138.8 ± 14.6	140.8 ± 16.8	134.7 ± 10.3	135.8 ± 11.7	136.2 ± 14.4 *	135 ± 5

EOT, end of treatment; STAIC, State-Trait Anxiety Inventory for Children; SD, standard deviation; TSCS:2, Tennessee Self-Concept Scale:2; ^‡^ Data are presented as means ± SD; * statistically significant compared to baseline (*p* ≤ 0.05).

**Table 9:** Comparisons of change (Δ) in the psychometric results at baseline and post-intervention, between groups.

Variables		Intervention (*n* = 22)	Control (*n* = 9)	Mean Difference (SED)	95% CI
		Δ (Mean ± SD)	Δ (Mean ± SD)		
STAIC	Absence of stress	−2.6 ± 2.8	−0.8 ± 5.0	−1.8 (1.4)	(−4.7 to 1)
	Presence of stress	−2.4 ± 3.1	0.1 ± 3.3	−2.5 (1.3)	(−5.1 to 0.1)
	State anxiety	−5.0 ± 2.7	−0.7 ± 2.6 ^††^	−4.3 (1.1)	(−6.5 to −2.2)
	Trait anxiety	−3.7 ± 6.7	−0.8 ± 8.0 ^†^	−2.9 (2.8)	(−8.6 to 2.8)
TSCS:2	Physical appearance	4.1 ± 3.1	0.9 ± 2.8 ^†^	3.2 (1.2)	(0.7 to 5.7)
	Moral	3.2 ± 4.4	0.8 ± 2.9	2.4 (1.6)	(−0.8 to 5.7)
	Personal	3.7 ± 3.3	−0.6 ± 1.2 ^††^	4.3 (1.1)	(2.0 to 6.6)
	Family	3.5 ± 3.1	−0.7 ± 1.4 ^†††^	4.2 (1.1)	(2.0 to 6.4)
	Social	5.3 ± 4.3	−1.2 ± 1.8 ^†††^	6.5 (1.5)	(3.5 to 9.6)
	Academic	2.6 ± 3.0	−1.2 ± 2.2 ^††^	3.8 (1.1)	(1.5 to 6.1)
	General self-perception	22.5 ± 15.6	−3.0 ± 4.7 ^†††^	25.5 (5.3)	(14.6 to 36.4)

CI, confidence intervals; STAIC, State-Trait Anxiety Inventory for Children; SD, standard deviation; SED, standard error of difference; TSCS:2, Tennessee Self-Concept Scale:2; statistically significant compared to intervention: ^†††^ *p* ≤ 0.001; ^††^ *p* ≤ 0.01; ^†^ *p* ≤ 0.05.

## Data Availability

The raw data supporting the conclusions of this article will be made available by the authors upon request.
